# Cryoablation and immune synergistic effect for lung cancer: A review

**DOI:** 10.3389/fimmu.2022.950921

**Published:** 2022-10-27

**Authors:** Yulong Tian, Xingshun Qi, Xin Jiang, Liqi Shang, Ke Xu, Haibo Shao

**Affiliations:** ^1^ Department of Interventional Radiology, The First Hospital of China Medical University, Shenyang, Liaoning, China; ^2^ Department of Gastroenterology, Northern Theater General Hospital, Shenyang, Liaoning, China

**Keywords:** cyroablation, immunotharapy, synergistic effect (combined treatment), NSCLC, lung cancer

## Abstract

The preferred treatment for lung cancer is surgical resection, but a large number of patients are not suitable for surgical resection in clinic. CT-guided cryoablation and immunotherapy can play an important role in patients with advanced lung cancer who are ineligible for surgery. CT-guided cryoablation has been widely used in the clinical treatment of lung tumors due to its advantages of less trauma, fewer complications, significant efficacy and rapid recovery. Cryoablation can not only cause tumor necrosis and apoptosis, but also promote the release of tumor-derived autoantigens into the blood circulation, and stimulate the host immune system to produce a good anti-tumor immune effect against primary and metastatic tumors. Since the study of immune checkpoint inhibitors has proved that lung cancer can be an immunotherapeutic response disease, the relationship between cryoablation and immunotherapy of lung cancer has been paid more attention. Therefore, we reviewed the literature on cryoablation for lung cancer, as well as the research progress of cryoablation combined with immunotherapy.

## 1 Introduction

Lung cancer is a serious threat to human health and the main cause of cancer-related death (1). About 1.59 million people worldwide die each year from lung cancer-related diseases (2). Non-small cell lung cancer (NSCLC) is one of the most common lung malignancies, and most of NSCLC is at an advanced stage at the time of diagnosis, which is not suitable for surgical resection (3). Chemotherapy and radiotherapy remain the two main treatments for patients with advanced lung cancer (4). Median overall survival (OS) was 8-12 months and 1-year survival was ~40% in patients treated with platinum-based two-drug regimen (5). Targeted therapy and immunotherapy improved survival benefit in lung cancer patients compared with chemorotherapy, but only patients with sensitive mutations or high expression of immune checkpoint proteins benefited from both treatments (6). How to improve the curative effect of immunotherapy becomes the bottleneck of further lung cancer treatment (7). Cryoablation as an alternative treatment for NSCLC that cannot be surgically resected (8). Cryoablation has been used in solid tumors such as liver cancer and pancreatic cancer with less trauma, quick recovery and good tolerability. In addition, cryoablation leads to tumor cell necrosis, which can further release antigen and increase the body’s ability to respond to antigen-antibody, thus achieving synergistic effect with immunotherapy (9, 10). This study will review the research progress of cryoablation and immunotherapy for lung cancer.

## 2 Cryoablation

Cryotherapy is one of the earliest minimally invasive ablation techniques in human history, which originated in the middle of the 19th century. In 1845, James Arnott, a British doctor, pioneered the modern era of cryotherapy by treating tumors with frozen saline water as low as -24°C (11). In 1993, the Joule-Thomson Principle, based on physics, was first developed in the world (12). It states that when gas is sprayed from a higher pressure area into a lower pressure area through a small hole, it will be throttled. The temperature of argon will drop after being throttled, while the temperature of helium will rise after being throttled. The Cryocare Surgical System uses high-pressure argon gas at room temperature as refrigerant and high-pressure helium gas as heat to treat tumors using the Joule-Thomson principle (13). Cryoablation has been used to treat liver tumors in the operating room setting for more than three decades (14). Currently, cryoablation has been widely used in the treatment of lung cancer, pancreatic cancer and other solid tumors (15).

### 2.1 Biological basis of cryoablation

The destruction of target tissues by cryoablation includes immediate and delayed effects, the former including intracellular and extracellular crystallization, and the latter including microvascular embolization and immune promotion. Under mild hypothermia, the death of target tissues mainly depends on the process of apoptosis.

#### 2.1.1 Direct killing effect on tumor cells

In the initial cooling stage, when the tissue temperature drops to minus 20°C, extracellular crystallization is gradually generated, which increases the concentration of extracellular solute, and the resulting osmotic pressure difference promotes the flow of intracellular water to the extracellular, resulting in cell dehydration and dysfunction. When the temperature drops below -40 °C, ice crystals can form in cells and directly damage organelles such as mitochondria and endoplasmic reticulum, thereby promoting cell death (16).

#### 2.1.2 Promoting the apoptosis of tumor cells

Apoptosis refers to the spontaneous and orderly death of cells controlled by genes in order to maintain homeostasis. Different from cell necrosis, apoptosis is not a passive process, but an active one, which involves the activation, expression and regulation of a series of genes. It is not a phenomenon of self-injury after cryoablation, but a death process that is actively fought for in order to better adapt to the living environment (17).

#### 2.1.3 The influence on the immune function of the body

After cryoablation, the body can produce anti-tumor antibody to inhibit secondary tumor growth and metastasis. The remaining frozen tumors were surrounded by reactive granulation and new capillaries, which were rich in tumor-infiltrating lymphocytes, plasma cells, monocytes and macrophages.

**Figure 1:**
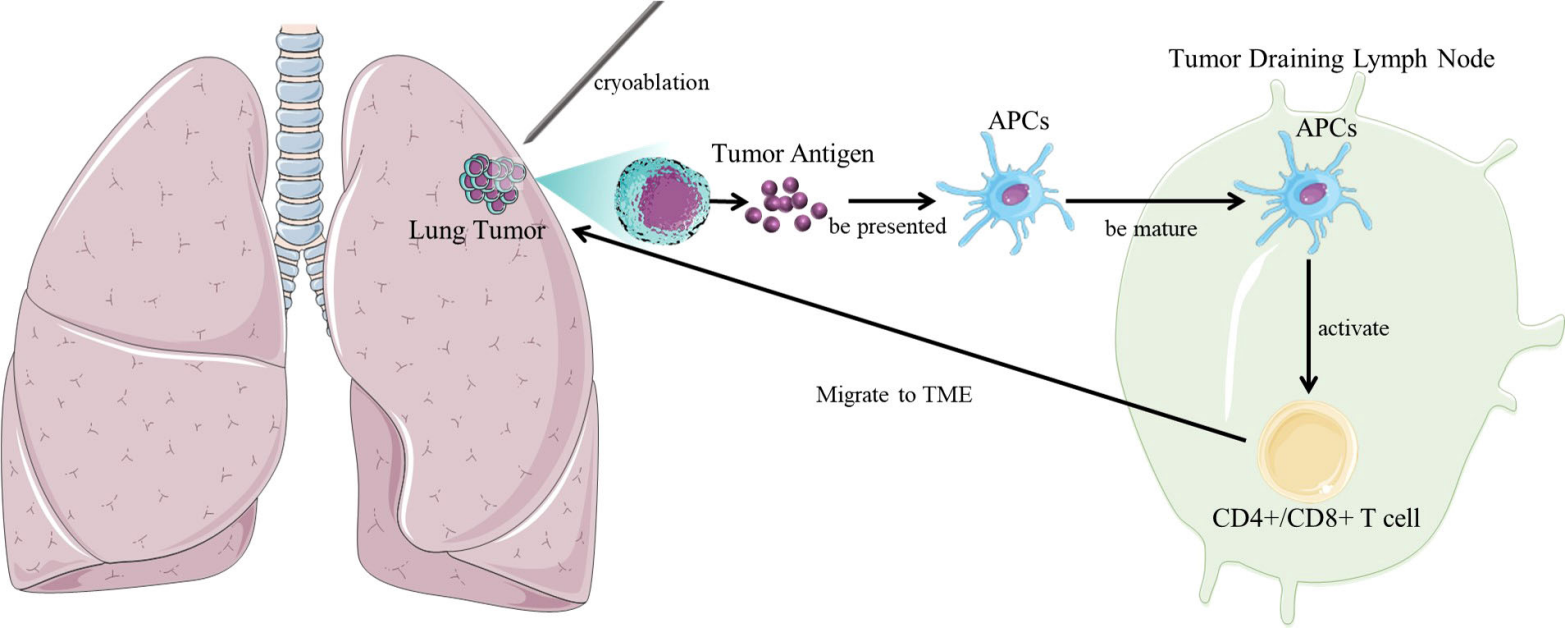
The interaction between tumor and immune system in cryoablation. In the mechanism of cryoimmunotherapy, frozen tumor cells release tumor antigens, which are taken up by immature myeloid dendritic cells (DCs). In the case of necrotic cell death following cryoablation, dendritic cells mature and migrate to tumor-draining lymph nodes (TDLNs), where they activate tumor-specific T cells and then migrate back to the tumor site. Achieve the synergistic effect of enhancing the body’s own immune function.

After cryoablation of the tumor, tumor antigen is activated and exposed antitumor cytotoxic antibody produces and induces cytotoxic T cell immunity, which enhances the anti-tumor immune response of patients, thereby eliminating residual or metastatic lesions, reducing or preventing recurrence (**Figure 1**). Therefore, theoretically cryoablation can prolong the survival of patients (18).

#### 2.1.4 Abscopal effect

The abscopal effect was first reported by Mole (19) in 1953. Abscopal effect refers to the phenomenon that can induce distant tumor regression after target tumor cryoablation (20). Cryoablation can promote tumor necrosis and release tumor antigen. The release of tumor antigen shows that the immune system is activated by tumor vaccine in situ. The tumor microenvironment changes from an immune desert state to an inflammatory immune infiltration state, which greatly promotes the anti-tumor immunity of the body. Even in a few cases, the abscopal effect can be observed (21). The abscopal effect makes its original local therapeutic effect play a systemic anti-tumor effect. However, the proportion of abscopal effect induced by cryoablation alone is low, which can only be seen in some case reports (22). With the advent of the era of tumor immunotherapy, especially the application of immunoassay point blockers, the abscopal effect of cryoablation has a breakthrough trend (23). But the mechanism of abscopal effect is still unclear. Theelen W et al. (24) confirmed the existence of radiation abscopal effect at the clinical level for the first time with statistically different potency. The mechanism is that the activated immune cells are mainly CD8+T cells that specifically kill distant tumor cells, leading to the occurrence of abscopal effect, and the addition of immune drugs is believed to amplify the effect.

## 3 Cryoablation in the treatment of lung cancer

The development of argon based cryoablation systems has greatly reduced the diameter of cryotherapy applicators (25). This makes transcutaneous application of the technique in other disease sites more feasible, as collateral damage to the skin and structure along the insertion path is minimized. Two types of percutaneous argon-based cryoablation devices are available on the market. They are Cryohit cryotherapy system and Endocare cryotherapy system (26). These systems allow the placement of 1 to 8 individual cryoprobes. The diameter of each cryoprobe is 1.5 to 2.4mm. In these systems, local tumor necrosis is usually achieved by a freeze-thawing-freeze cycle of a single probe location. The combination of multiple cryoneedles can shorten the ablation time and create a larger ablation area. A new freeze-thawing model was used during lung tumor cryoablation: 5 min freezing, 3 min thawing, 10 min freezing, 3 min thawing, 10 min freezing. This new model is used to generate interstitial fluid in adjacent lung tissue, which further forms a ball of ice into the lung, thereby increasing the ablation range of lung tumors (27).

### 3.1 Image guidance and range evaluation of cryoablation

CT scans are usually performed after cryoablation to measure changes in low density within the target tumor and are used to estimate the size of the ablation area. The periphery of the frozen zone may not reach cytotoxic temperature. Therefore, a margin of 3-7mm was subtracted from the diameter of the low-density ablation area to better approximate the true volume of tissue necrosis. The low-density hockey ball on CT images can be directly used to compare the relationship between the ablation area and tumor margin. This allows the operator to be more confident in treating tumor tissue adjacent to important organs or blood vessels and to measure cytotoxic ice margins. Multiple cryoablation needles must be used to treat large tumors (15, 28, 29).

### 3.2 Advantages and disadvantages of cryoablation for lung tumors

Compared with thermal ablation, the advantages of cryoablation include the following aspects: the combination of multiple cryoablation needles can obtain larger tumor ablation volume. The ablation area is highly visible under CT scan, which makes the ablation range more accurate and effective. Patients are well tolerated due to the analgesic effect of cryoablation. Cryoablation is a safer option for lesions near blood vessels or bronchus because it preserves collagen and cellular structures in frozen tissue. One disadvantage of cryoablation is puncture bleeding. The latest cryoablation systems allow additional radiofrequency heating of the frozen probe shaft, allowing thermal solidification of the probe prior to probe removal. Another disadvantage of cryoablation compared to thermal ablation is the longer procedure time required to produce adequate tumor coverage.

### 3.3 Clinical studies of cryoablation for lung tumors

Several large-scale studies have evaluated cryoablation for intrathoracic lesions, suggesting that cryoablation is an effective and safe treatment (4, 30). The potential efficacy of cryotherapy is illustrated by the following (**Table 1**):

**Table 1:** Clinical study of cryoablation for lung tumors.

Year	References	Tumor types	Number of patients	Observation indicators and results	Complication	Follow-up time
2013	Pusceddu, C. et al. (31)	NSCLC and metastatic lung malignancy	N=32	Technical success was 92%.	Pneumothorax occurred in 21% cases and 3% cases asymptomatic small pulmonary hemorrhage,	6 months
2013	Yashiro, H. et al. (32)	Primary lung malignancy and metastatic lung malignancy	N=71	Local tumor progression occurred in 50 tumors (23.8%). One-, 2-, and 3-year local progression-free rates were 80.4%, 69.0%, and 67.7%, and technique effective rates were 91.4%, 83.0%, and 83.0%.	To reduce the risk of bleeding and pneumothorax, we removed the coaxial needle after plugging with fibrin glue along the tract through the sheath.	15 months.
2015	Moore,W. et al. (33)	Stage I NSCLC	N=45	The 5-year survival rate was 67.8%, the cancer-specific survival rate was 56.6%, and the 5-year progression-free survival(PFS) rate was 87.9%. The recurrence rate was 36.2%.	Major complications occurred in 6.4% of patients, including two cases of hemoptysis and a prolonged placement of a chest tube requiring mechanical sclerosis in one patient.	5 years
2015	Kim, K. Y. et al. (34)	Ground-glass opacity (GGO)	N=1	Cryoablated successfully without recurrence.	No major procedure-related complications	6 months.
2018	Lyons, G. R. et al. (35)	Primary lung malignancy and metastatic lung malignancy	N=42	The recurrence was 11.4%, 11.4%, and 38.1% at 1, 2, and 3 years after cryoablation, respectively.	Pneumothorax occurred in 19 cases (33.9%), 7 (12.5%) requiring a chest tube.	4 years
2018	Gao, W. et al. (36)	Stage IIIB/IV NSCLC	N=22	The technique effectiveness of 100%. The one-year survival rate of 81.8% and progression-free rate of 27.8% was obtained.	Twelve cases suffered from chest pain, low-grade fever or general malaise, while four cases had pneumothorax. And three cases with hemoptysis and two cases with pleural effusion were observed.	1year
2019	Liu, S. et al. (37)	Ground-glass opacity (GGO)	N=14	Cryoablated successfully without recurrence.	Pneumothorax occurred in 3 cases, lung volume was compressed to approximately 5%.Blood−stained sputum was noted in 5 cases.	24 months,
2020	Das, S. K. et al. (38)	Stage IIIB/IV NSCLC	N=45	PFS was 10 months. OS time was 27.5 months.	Pneumothorax occurred in 17 cases (37.8%). 5 cases (11.1%) required the use of a chest tube drainage. Intrapulmonary hemorrhage occurred in 11 cases (24.4%).	19.5 months.
2021	Iezzi, R. et al. (39)	Primary lung malignancy and metastatic lung malignancy	N=20	Technical success was100%.	Pneumothorax occurred in 4 cases, 3 cases required the use of a chest tube drainage.	13 months.

#### 3.3.1 Cryoablation for NSCLC (T1N0M0)

Moore,W. et al. (33) retrospectively evaluated the effect of cryoablation on T1N0M0 NSCLC from 2006 to 2011. Forty-five patients were treated with cryoablation and followed up for 5 years. Local and regional recurrence rates and complications were monitored. The 5-year survival rate was 67.8%, the 5-year cancer-specific survival rate was 56.6%, and the 5-year progression-free survival rate was 87.9%. Local and regional combined recurrence rate was 36.2%. Major complications including 2 cases of hemoptysis and 1 case of mechanical sclerosis due to long-term placement of chest tubes. There were no deaths in the first 30 days after treatment.

#### 3.3.2 Cryoablation for advanced NSCLC

Lyons, G. R. et al. (35) report on the safety and efficacy of cryoablation in patients with lung tumors. From 2012 to 2016, 42 patients were treated with cryoablation. The average diameter of the nodules was 1.6cm. 13 patients were primary lung malignancies, and 54 patients were secondary lung malignancies. The average age was 68.1 years, and the male to female ratio was 1.3 to 1. The mean radiographic follow-up was 326 days. Nineteen patients developed pneumothorax and 7 required thoracic drainage. Local tumor recurrence/residual in 6 cases. The estimated marginal probability of local recurrence at 1, 2 and 3 years after ablation was 11.4%, 11.4% and 38.1%, respectively.

Pusceddu, C. et al. (31) reported experience with cryoablation for primary and secondary lung tumors.32 cases (24 males, 8 females; mean age 67) were not suitable for surgical removal of lung cancer. All the cryoablation treatments were successfully completed. There were no surgery-related deaths. The incidence of complications was 21% pneumothorax and 3% asymptomatic minor pulmonary hemorrhage, respectively. At 1, 3 and 6-month follow-up CT scans, technical success rates for treating lesions were 82%, 97% and 91%, respectively. The technical success rate was 92%. Yashiro, H. et al. (32) reported data on percutaneous cryoablation of lung tumors. The mean maximum diameter of the lesions in 71 patients was 12.8mm. Kaplan-meier method was used to evaluate local tumor progression rate and technical efficacy. The median follow-up time was 454 days, and 50 patients developed local tumor progression. The 1-year, 2-year and 3-year local progress-free rates were 80.4%, 69.0%, 67.7%, and the technical effective rates were 91.4%, 83.0%, 83.0%, respectively. Gao, W. et al. (36) reported on percutaneous cryoablation for the treatment of advanced non-small cell lung cancer after failure of radiotherapy and chemotherapy. A total of 22 patients with stage IIIB/IV advanced NSCLC after failure of radiotherapy and chemotherapy were included. To evaluate the efficacy and follow-up after cryoablation. The main technical effective rate was 100% after 1-month follow-up. At 3 months, 4 of 31 lesions had local tumor progression. The 1-year survival rate was 81.8% and the progression-free rate was 27.8%. Das, S. K. et al. (38) reported data from a study of cryoablation for stage IIIB/IV NSCLC. Forty-five patients with lung cancer underwent cryoablation. Progression-free survival (PFS), overall survival (OS), and adverse events (AEs) were observed. PFS was 10 months. OS time was 27.5 months. The number of ablation needles and tumor size were associated with the risk of pneumothorax and intrapulmonary hemorrhage. Yang, W. et al. (40) reported on a multicenter randomized controlled trial (RCT) evaluating the efficacy and safety of cryoablation for stage III-IV NSCLC. The primary efficacy endpoints were ice hockey coverage rate (ICR) and disease control rate (DCR) at 1 month after treatment. Forty-one patients received Argon-Helium Cryoablation with ICRs of 98.66%. DCR is 95%. The complication rate was 30%.

#### 3.3.3 Cryoablation for pulmonary ground glass nodules

Kim, K. Y., et al. (34) reported preliminary experience of cryoablation of pure GGO residue after repeated surgical resection in a patient with multiple GGO. Cryoablation of 5mm pure GGO in the left lower lobe was successful, and there was no recurrence after 6 months of follow-up. Liu, S. et al. (37) evaluated the safety and feasibility of cryoablation for pulmonary ground glass nodules. Fourteen patients with pulmonary GGO underwent cryoablation. Adverse events, lung function, and therapeutic outcomes after cryoablation were assessed. No serious complications occurred in all patients, and lung function recovered to 95% one month after cryoablation. GGO appeared to be successfully ablated in all patients at 24 months of follow-up with computed tomography.

## 4 Cryoimmunotherapy for lung tumors

### 4.1 Mechanisms of lung tumors in cryoimmunotherapy

Alteber, Z. et al. (41) reported that local administration of immature dendritic cells (DCs) enhances the immune response induced by cryoablation. The lung cancer model of murine Lewis lung cancer d122-LUC-5.5 was used. The tumor was treated with local cryotherapy combined with immunotherapy. Clinical outcomes were assessed by monitoring tumor growth, metastasis to distant organs, overall survival, and protection against tumor recurrence. The nature of the induced T cell response was analyzed. Combined cryoimmunotherapy can reduce tumor growth, reduce metastasis rate and significantly prolong survival. In addition, this treatment induced antitumor memory and protected the mice from rechallenge. The underlying mechanisms are the generation of tumor-specific Type 1 T cell response, subsequent induction of cytotoxic T lymphocytes, and the generation of systemic memory. Zhang, M. et al. (42) reported a study of dendritic cells (DCs) combined with the immune adjuvant cytidine guanidine oligodeoxynucleotide (CPG-ODN) combined with cryoablation for lung cancer. Mice with Lewis lung cancer (LLC) were cryoablated and cultured dendritic cells were injected into the peritumoral region. Subsequently, experimental animals were given CPG-ODN at 6 h, 12 h, and 24 h after DCs injection. The changes of T cell subsets were determined. The results showed that the proportion of CD4+ and CD8+ T cells in cpG-ODN group increased 12 h after DCs injection. Conclusion CpG-ODN is closely related to the efficacy of cryoablation, dendritic cell and immune adjuvant combined therapy.

### 4.2 Clinical studies of cryoimmunotherapy for lung tumors

Yuan Y. et al. (43) conducted a retrospective study of 21 patients with metastatic non-small cell lung cancer (NSCLC). To evaluate the efficacy of combined cryotherapy, and dendritic cell-activated cytokine induced killer cell (DC-CIK) immunotherapy. The overall survival (OS) from diagnosis of metastatic NSCLC to death was assessed during a 5-year follow-up period. Patients who received combined cryotherapy had an OS of 20 months, significantly longer than those who did not receive cryotherapy at an OS of 10 months. Lin, M. et al. (44) evaluated the safety and clinical efficacy of cryotherapy combined with exogenous NK cell immunotherapy in the treatment of advanced non-small cell lung cancer (NSCLC). Sixty NSCLC patients were enrolled and divided into two groups: the cryoablation group alone and the cryoablation combined with allogeneic NK cells group. Changes in immune function, quality of life, and clinical response were assessed. It was found that allogeneic NK cells combined with cryotherapy had a synergistic effect on advanced NSCLC, which not only enhanced the immune function and improved the quality of life of patients, but also significantly improved the response rate (RR) and disease control rate (DCR) compared with the cryotherapy group. Takaki, H. et al. (45) explored changes in peripheral blood T cells after percutaneous tumor cryoablation. A total of seven lung cancer patients were included in the study. Peripheral blood samples were collected before cryoablation and 14 days after ablation. The number of cytotoxic T cells (CTL), type 1 (Th1), type 2 helper T cells (Th2) and regulatory T cells (Treg) in peripheral blood were detected by flow cytometry. The results showed that CTL population and CTL/Treg ratio in peripheral blood increased by 28.9% and 21.3% on average. Th1/Th2 ratio remained unchanged after ablation. Feng, J. et al. (46) investigated the clinical safety and efficacy of argon helium cryoablation combined with nivolumab in the treatment of advanced NSCLC. 64 patients with advanced NSCLC were divided into 2 groups on average in a retrospective study. All patients underwent argon helium cryoablation combined with nivolumab or cryoablation alone at a single center. Short-term efficacy, adverse reactions, immune function, tumor marker cytokeratin 21-1 (CyFRA21-1), carcinoembryonic antigen (CEA), neuron specific enolase (NSE) and circulating tumor cell (CTCs) levels were compared between 2 groups. Patients in the nivolumab combining with cryoablation group showed significant improvement in immune function and short-term efficacy. The levels of CTC, tumor marker CyFRA21-1 and NSE were significantly decreased in lannierumab group (**Table 2**).

**Table 2:** Cryoimmunotherapy for lung tumors.

Year	References	Tumor types	Sample size	Combined therapies	Observation indicators and results
2013	Yuan, Y. et al. (43)	Metastatic NSCLC	N=21	Dendritic cell-activated cytokine-induced killer cells (DC-CIK) immunotherapy.	OS was 20 months, significantly longer than those who did not receive cryotherapy at an OS of 10 months.
2017	Lin, M. et al. (43, 44)	Advanced NSCLC	N=60	Allogenic NK cell immunotherapy	Combined treatment has a synergistic effect, which not only enhancing the immune function of patients, improving the quality of life, and increasing the response rate (RR) and disease control rate (DCR).
2021	Feng, J. et al. (46)	Advanced NSCLC	N=64	Nivolumab	Combined treatment had a significant improvement in immune function and short-term efficacy. Levels of CTCs and tumor markers CYFRA21-1 and NSE were reduced.

## 5 Conclusion

Cryoablation has proven to be a successful measure of local control of primary and secondary lung cancers. In most cases, it is appropriate for patients with early-stage tumors or who are not suitable for surgery. The synergy of local ablation with systemic therapy is one of the most exciting developments in interventional oncology. In particular, it is considered that cryoablation provides the immune system with tumor-associated antigens that induce immune-specific activation against tumor cells. Further high-quality studies are needed to determine the immunological effects of percutaneous cryoablation and the efficacy of cryoablation combined with immunotherapy for lung cancer.

## Author contributions

YT: Conceptualization, data curation, formal analysis, resources, writing—original draft, and writing—review and editing. XQ: Methodology, supervision, and writing—review and editing. XJ: Formal analysis, investigation, and validation. LS: Formal analysis, resources. KX: Funding acquisition, investigation, and supervision. HS: Funding acquisition, project administration and supervision. All authors contributed to the article and approved the submitted version.

## Funding

National Natural Science Foundation of China (NO.82072037). The corresponding author, HS, is the chief expert of the project.

## Conflict of interest

The authors declare that the research was conducted in the absence of any commercial or financial relationships that could be construed as a potential conflict of interest.

## Publisher’s note

All claims expressed in this article are solely those of the authors and do not necessarily represent those of their affiliated organizations, or those of the publisher, the editors and the reviewers. Any product that may be evaluated in this article, or claim that may be made by its manufacturer, is not guaranteed or endorsed by the publisher.
